# Breaking the Cycle of Addiction: Prevention and Intervention With Children of Alcoholics

**Published:** 1997

**Authors:** Ann W. Price, James G. Emshoff

**Affiliations:** Ann W. Price, M.A., is a doctoral candidate and James G. Emshoff, Ph.D., is an associate professor at the Department of Psychology, Georgia State University, Atlanta, Georgia

**Keywords:** children of alcoholics, behavioral problem, AOD dependence, prevention program, prevention model, program evaluation, group participation, intervention, risk factors, mutual help and support group, school based prevention, AOD education, coping skills, social behavior, social support, basic research, prevention through education, literature review

## Abstract

Children of alcoholics (COA’s) are at increased risk for behavioral and emotional problems, including alcoholism. Research has helped guide the design of prevention and intervention programs aimed at reducing this risk. Currently, most such programs for COA’s use a short-term, small-group format, often conducted within schools. Broad-based community programs are another promising option, but have not been sufficiently studied. Generally, interventions include alcoholism education, training in coping skills and social competence, social support, and healthy alternative activities. Increased interaction between basic research and intervention may lead to improved services for COA’s.

Children of alcoholics (COA’s) are at increased risk for a wide range of behavioral and emotional problems, including addiction to alcohol and other drugs (AOD’s), depression, anxiety, school failure, and delinquency (Adger 1997; Emshoff and Anyan 1989; Sher 1991). Prevention and intervention efforts attempt to reduce this risk by modifying risk-associated factors. In general, prevention programs target children because of the behavior of an adult caregiver, rather than because of the child’s own behavior. Intervention programs, however, usually target children who have begun to exhibit symptoms themselves, such as depression, poor academic performance, or problems getting along with their peers.

Several types of programs have been developed to assist COA’s. Although a program may focus primarily on either prevention or intervention, most programs include elements of both. Therefore, this article discusses both types of programs somewhat interchangeably. In addition, the discussion primarily focuses on programs provided in group settings.

## Prevention Models

Primary prevention focuses on children who have not exhibited specific problems but who may be at risk because of genetic or environmental factors or both. Secondary prevention (i.e., intervention) is targeted toward children who already exhibit behaviors predictive of later AOD use. Finally, the goal of tertiary prevention (which is analogous to treatment) is to help children who are already involved with AOD’s and to prevent further deterioration of their behavior.

Albee (1978) suggests that the risk for behavioral problems is increased by exposure to stress and reduced by social support, social competency (i.e., social skills), and self-esteem. Therefore, the goals of primary prevention with COA’s should include stress reduction and the development of self-esteem, social competence, and a strong social support system.

Other primary prevention models take a different approach. For example, the “distribution of consumption” model proposes to reduce the general public’s consumption of alcohol by limiting its availability. This theoretically would reduce the number of problem drinkers and consequently the number of children exposed to alcohol problems in the family. This strategy involves raising the drinking age, limiting “happy hours,” and increasing the price of alcoholic beverages or limiting the hours of their sale. This approach will not be discussed here, however, because it does not specifically target COA’s.

Another model, called the “sociocultural model,” focuses on education and on enhancing children’s competencies through information, values clarification (i.e., examining values regarding alcohol), and skill-building techniques. The goal of this approach is to teach children to moderate their drinking and avoid later alcohol problems. Sociocultural programs can be implemented throughout the community or may be targeted via schools, recreational activities, or physicians’ offices (Williams 1990).

## Screening and Identification

Many COA’s never receive intervention services. COA’s are usually identified incidentally when the child’s parent enters alcoholism treatment. This type of identification is ineffective in reaching the majority of COA’s, because most alcoholics never receive treatment (Emshoff and Anyan 1989). In addition, few children seek help voluntarily, because family denial puts pressure on the child to keep the family’s secret (Dies and Burghardt 1991).

Identification of COA’s, therefore, requires a process of active screening. To this end, Dies and Burghardt (1991) describe certain behavior patterns that suggest a child may have an alcoholic parent. Some of these behaviors may reflect lack of parental supervision, such as frequent tardiness or absence from school or carelessness in dress or personal hygiene. Other possible indicators of COA status include emotional instability, immaturity, conflict with peers, isolation from other children, academic problems, or physical complaints (e.g., headaches and stomach aches). Many people who work with children are not trained to recognize these subtle signs; in addition, these signs are not specific to COA’s. Therefore, researchers have developed questionnaires to identify COA’s who do not display obvious behavior problems (Dies and Burghardt 1991).

One commonly used screening instrument is the CAGE, a set of four questions regarding the respondent’s concern over his or her own drinking behavior. The Family CAGE is slightly reworded to reflect a respondent’s concern for the drinking habits of a relative. This questionnaire is intended to screen for, not diagnose, family alcoholism; a positive finding on the Family CAGE should be followed by a complete diagnostic assessment (see box).

**The Family CAGE: An Alcoholism Screening Test**

The CAGE is perhaps the most widely used screening test for alcoholism. This tool has been adapted to reflect concern for a parent’s drinking through the following four questions:

1. Do you think your parent needs to **C**UT down on his/her drinking?
2. Does your parent get **A**NNOYED at comments about his/her drinking?
3. Does your parent ever feel **G**UILTY about his/her drinking?
4. Does your parent ever take a drink early in the morning as an **E**YE opener?

Another useful questionnaire is the Children of Alcoholics Screening Test (CAST) (Jones 1982; Sheridan 1995), designed to identify both young and adult children of alcoholics. The 30-item instrument probes the respondent’s attitudes, feelings, perceptions, and experiences related to the drinking behavior of the respondent’s parents. A shorter version of CAST has also been developed. Because of time constraints and the fact that many school-based programs are run by teachers rather than psychologists, such measures are not routinely used to identify COA’s in the school environment.

## Prevention Groups

Most programs for COA’s are delivered in group settings. Group programs reduce COAs’ feelings of isolation, shame, and guilt (Dies and Burghardt 1991) while capitalizing on the importance to adolescents of peer influence and mutual support (Dies and Burghardt 1991). Groups may be structured and closed-ended, with a specific beginning and end-point, or open-ended, with participants joining and leaving the group as they feel the need.

Groups may be directed at the general population of COA’s, as in broad-based community prevention programs, or targeted at specific high-risk groups, such as abused or neglected children as well as youth with academic problems or gang affiliations. These groups are readily identified and contain a large percentage of COA’s. Prevention and intervention services can be offered to high-risk COA’s as part of comprehensive social service programs aimed at those populations.

### Alateen

Alateen is an example of a community-based self-help program for COA’s based on the 12-Step approach of Alcoholics Anonymous. Alateen generally meets in public settings, such as churches or community centers. Little data exist on the effectiveness of Alateen. In one study, COA’s participating in Alateen had more positive scores than nonparticipating COA’s on a scale measuring mood and self-esteem, factors affecting risk for behavioral problems, including alcohol misuse (Hughes 1977). Conversely, in a study of 4- to 16-year-old sons of alcoholics, Peitler (1980) found that group counseling had more positive effects than did Alateen in improving self-worth. Unfortunately, not enough empirical evidence exists to draw any firm conclusions about the effectiveness of Alateen.

### School-Based Groups

Children are available at schools for long periods of time and in large numbers; therefore, educational institutions are logical settings for intervention efforts. Behavior problems potentially indicating parental alcoholism can be most readily recognized in school (Dies and Burghardt 1991). An added benefit is that COA programs within schools have ready access to needed information and services. Finally, children and adolescents may find it embarrassing to attend programs at an outside agency or treatment center, particularly in settings that may have a negative stigma attached (e.g., mental health centers).

School curricula often include information about AOD’s and their impact on the family. These AOD education classes provide a valuable opportunity for teachers to observe possible signs of parental alcoholism. For example, COA’s may be extremely negative or apprehensive about alcohol and drinking or may exhibit changes in attendance patterns or interest levels during AOD education. Although schools appear to be logical prevention settings, few school-based programs designed specifically for COA’s have been described (Dies and Burghardt 1991). Therefore, the types and prevalence of such programs are not known.

## Program Content

Although there are several types of intervention programs, some strategies are common to most programs. Among these strategies are training in social competency and coping skills, as well as providing information, social support, and alternatives to AOD use. These strategies have been developed for prevention efforts with diverse populations, but are applied (and sometimes adapted or customized) to groups of COA’s.

The content of COA prevention and intervention programs is often based on social cognitive theory (Bandura 1986). The goals of such programs are to reduce children’s stress, increase their social support system, provide specific competencies and skills, and provide opportunities for increased self-esteem. Social cognitive theory emphasizes techniques such as role playing, modeling, practice of resistance skills, and feedback. Role playing allows the child to rehearse common situations such as riding in the car with a drunk parent. Through modeling, participants learn appropriate behavior (e.g., effective communication skills) by observing group leaders and peers. Resistance skills help children cope with peer pressure to drink. Both the group leader and participants provide the child with positive feedback to reinforce and encourage newly acquired skills. These techniques have contributed to significant reductions in the use of cigarettes, alcohol, and marijuana in general prevention programs that target wider groups rather than COA’s specifically (Ellickson and Bell 1990; Pentz 1985). More research is needed to test these techniques with COA’s.

The influence of the child’s developmental stage must be considered during program design. For example, elementary school-aged children do not always have realistic perceptions of relationships and causal links and may believe that they are the cause of their parents’ drinking problem. During the middle school years, COA’s, as well as other children, often make decisions about using AOD’s themselves. In addition, the emergence of emotional or mental health problems is not unusual for many adolescents, including COA’s (West and Prinz 1987). Therefore, prevention efforts should focus on the preteen years.

Many COA’s who appear to be coping well are actually in a self-protective state of denial. Group facilitators should exercise patience and sensitivity as children adjust to their changing awareness about their parents’ drinking. Group leaders should also recognize that COA’s may become overly dependent on them and should be sensitive to the feelings of abandonment that children may experience when the group terminates.

### Information and Education

Most programs provide information about alcohol and alcoholism to help correct false expectancies. For example, COA’s often overestimate the positive effects of alcohol consumption on cognitive and social performance (Brown et al. 1987; Mann et al. 1987), thereby increasing their risk for excessive drinking.

Most programs promote the concept of alcoholism as a disease to help the child put the behavior of the alcoholic parent in perspective. For example, understanding the biological basis of alcoholism manifestations such as tolerance, blackouts, and withdrawal helps the child overcome misplaced self-blame and guilt about parental drinking. Finally, COA’s must learn that they are at risk for a variety of psychosocial problems, especially alcoholism. Research shows that COA’s who are aware of their risk status drink significantly less than COA’s who are unaware of their risk status (Kumpfer 1989).

### Competencies and Coping Skills

Competencies are skills that help children cope with stress, thereby reducing their risk for alcoholism and other psychosocial problems. Most programs teach specific emotion-focused and problem-focused coping skills (Nastasi and DeZolt 1994; Folkman 1984). Emotion-focused coping is a process by which the child seeks social support or uses strategies such as distancing or reframing the negative aspects of the situation to emphasize the positive aspects. For example, the child’s inability to control parental drinking may be offset by the knowledge that sources of help are available.

Problem-focused coping emphasizes the problems of living in an alcoholic home, such as having to explain unusual parental behavior to friends. In addition, this approach attempts to enhance decisionmaking, problem-solving, and communication skills, as well as the ability to resist peer pressure to drink, as discussed earlier under “Program Content.” Emotion-focused and problem-focused skills are not mutually exclusive, and children who learn both skills are better equipped to manage their lives.

### Personal-Social Competencies

Personal-social competencies can improve COA functioning despite exposure to stress (Albee 1978; Dohrenwend 1978). Such competencies include the ability to establish and maintain intimate relationships, express feelings, and solve problems (Nastasi and DeZolt 1994). These skills can be enhanced by buttressing the COA’s self-esteem and self-efficacy (i.e., the belief that one can perform a particular task).

Social support arises naturally out of participation in group treatment. In the group setting, children often learn for the first time that other children have problems similar to theirs. Many children benefit from sharing their experiences and emotions in a safe environment with other children. Through mutual exchange, children learn survival skills from the experiences of their peers, gain practice in expressing feelings, and build their social support networks.

Many COA’s attempt to achieve perfection in everything they do as a means of acquiring self-esteem. This sets the stage for inevitable failure. Therefore, interventions often emphasize alternative ways to acquire self-esteem and self-efficacy, as discussed next.

### Alternative Activities

Alternative activities provide opportunities for COA’s to participate in activities that exclude alcohol, tobacco, and other drugs. Healthy alternative activities (e.g., sports, peer leadership training institutes, and programs such as Outward Bound) may help children build a sense of self-efficacy; increase self-esteem; provide a positive peer group; and increase life skills, such as problem-solving and communication. Programs may focus exclusively on alternative activities but preferably are part of a comprehensive prevention program.

## Evaluation Findings

The examples below are among the few prevention programs for COA’s for which evaluation data are available. These programs exemplify the prevention components discussed in this article.

### Stress Management and Alcohol Awareness Program

Roosa and colleagues (1989) developed a competency-building intervention called the Stress Management and Alcohol Awareness Program (SMAAP). SMAAP is an 8-week, school-based program for COA’s, focused on building self-esteem, providing alcohol-related education, and teaching emotion- and problem-focused coping strategies.

A recent revision of SMAAP (Short et al. 1995) attempts to correct children’s misconceptions about alcohol (Brown et al. 1987; Mann et al. 1987). The revised curriculum also includes additional practice using coping skills and provides a “personal trainer” who meets weekly with participants to reinforce the personal and social skills children learned through the program.

Recent evaluation showed that 9-to 11-year-old COA’s who participated in a SMAAP program were more likely than nonparticipant COA’s to report increased knowledge, social support, and emotion-focused coping behavior (Short et al. 1995). In addition, teachers reported increased problem-solving and social competence among participants. Teachers were not blind to group membership, however, raising the possibility that their expectancies may have influenced their judgment. The results also showed an unintended negative side effect: An overall significant increase occurred in the expectation that alcohol consumption can serve to reduce tension. Additional research is needed to clarify this finding, because past research indicates a significant relationship between positive alcohol expectancies and greater alcohol use by adolescents (Christansen et al. 1989; Mann et al. 1987). Finally, no differences in outcome occurred between groups that received or did not receive the Personal Trainer component of the program, possibly because the program did not last long enough for such differences to emerge.

### Students Together And Resourceful

The Students Together And Resourceful (STAR) program is designed to provide students with accurate information on alcoholism and its effects on the family as well as increase social competence skills. Group exercises are directed to help students recognize and express their feelings and to practice specific skills, such as problem-solving, decisionmaking, stress management, and alcohol-refusal skills.

Emshoff (1990) used a randomized design to compare COA’s participating in STAR with nonparticipant COA’s over a period of time. Results indicated that participants were successful in establishing stronger social relationships, a sense of control, and an improved self-concept. In addition, participants reported increases in the number of friends and in perceived social support. Participants also reported decreased loneliness and depression (Emshoff 1990).

### Strengthening Families Program

The Strengthening Families Program (SFP) provides training for parents, children, and families. Sessions for parents focus on AOD education, communication skills, and the use of reinforcement and other techniques to guide children’s behavior. The children’s social skills program includes sessions on feelings, anger management, problem-solving, communication, peer resistance, and AOD information. The family component provides the opportunity for families to practice their skills through structured play sessions. Typically the program is conducted in churches or community centers in eighteen 2- to 3-hour sessions. In a randomized, controlled trial, the program was found to reduce risk factors, increase resilience (i.e., competence when under stress), and decrease AOD use among children of AOD abusers (Kumpfer et al. 1996).

The basic SFP for 6- to 12-year-olds has been modified for a variety of cultural groups, including rural and urban African-Americans, Hawaiians, Hispanics, and rural preteens (Kumpfer et al. 1996). Evaluation studies showed that the basic program with minor cultural revisions was more effective than a substantially revised program. Therefore, researchers concluded that the core content of the program should be spared when cultural revisions are made. The National Institute on Drug Abuse has chosen SFP as one of three model AOD abuse prevention programs for dissemination by the Institute.

### Cambridge and Somerville Program for Alcoholism Rehabilitation

The Cambridge and Somerville Program for Alcoholism Rehabilitation (CASPAR), located in Somerville, Massachusetts, offers a range of prevention and intervention services, including programs for COA’s. Sessions are conducted by adult staff in school and community settings and by trained peer leaders in after-school groups in junior high schools (Davis et al. 1994). Basic groups provide general information about alcohol-related problems and are open to any child who wishes to attend. COA-specific groups concentrate on alcoholism in the family and strategies for coping.

DiCicco and colleagues (1984) compared COA’s in a COA-specific group with both COA’s and non-COA’s in basic alcohol education groups. Participants in the COA-specific groups reported greater willingness to confide their problems and feelings to other group participants than did COA’s or non-COA’s in basic groups. Nevertheless, more COA’s in the basic alcohol education group reported that they intended to drink differently and were drinking less as a result of participation compared with either COA’s in the COA-specific group or non-COA’s in the basic groups. In addition, COA’s in general appear more willing to attend the basic education group than the COA-specific group in order to avoid stigmatization. These results suggest that mixed groups of COA’s and non-COA’s may be a valuable option for reaching and helping children from alcoholic families (DiCicco et al. 1984).

## Conclusions

Despite their risk status, most COA’s are remarkably well adjusted (Nastasi and DeZolt 1994; Serrins et al. 1995; Sher 1991). Nevertheless, many children exhibit emotional and behavioral problems as a result of parental drinking. Improved research methods can guide intervention to prevent adverse outcomes from developing.

More stringent methods of program design and evaluation are needed to bolster research efforts. These methods include better sampling procedures, random assignment of subjects to treatment types, the use of untreated control groups for comparison, appropriate sample sizes, developmentally and culturally appropriate screening and diagnostic instruments, and more precise definitions of parental alcoholism.

Emshoff and Anyan (1989) called for the use of “action” research, a model that emphasizes an interactive relationship between research and intervention. This approach would emphasize evaluations of a subject’s functioning over time. As part of the action research model, the dissemination of evaluation results is an important step leading to improved services for COA’s. Evaluation data also indicate the need for future research, thus contributing to the continual cycle between basic and applied research.

Results of evaluation research suggest several appropriate levels of intervention and basic prevention program components. Basic AOD education should be included in public school curricula. Parental and family training are promising areas that have been shown to reduce child and adolescent risk factors (Dishion and Andrews 1995; Webster-Stratton et al. 1988). Comprehensive community programs that target social norms regarding AOD’s are another promising, yet underutilized, area. Preventive intervention programs should include the basic components of information and education, skill building in the areas of coping and social competence, social support, an outlet for the safe expression of feelings, and healthy alternative activities.
